# Precision autofocus in optical microscopy with liquid lenses controlled by deep reinforcement learning

**DOI:** 10.1038/s41378-024-00845-8

**Published:** 2024-12-24

**Authors:** Jing Zhang, Yong-feng Fu, Hao Shen, Quan Liu, Li-ning Sun, Li-guo Chen

**Affiliations:** 1 School of Mechanical and Electrical Engineering, Soochow University, No.8 Jixue Road, Suzhou City, Jiangsu 215000 China; 2 School of Computer Science and Technology, Soochow University, No.333 Ganjiang East Road, Suzhou City, Jiangsu 215006 China

**Keywords:** Optical sensors, Micro-optics

## Abstract

Microscopic imaging is a critical tool in scientific research, biomedical studies, and engineering applications, with an urgent need for system miniaturization and rapid, precision autofocus techniques. However, traditional microscopes and autofocus methods face hardware limitations and slow software speeds in achieving this goal. In response, this paper proposes the implementation of an adaptive Liquid Lens Microscope System utilizing Deep Reinforcement Learning-based Autofocus (DRLAF). The proposed study employs a custom-made liquid lens with a rapid zoom response, which is treated as an “agent.” Raw images are utilized as the “state”, with voltage adjustments representing the “actions.” Deep reinforcement learning is employed to learn the focusing strategy directly from captured images, achieving end-to-end autofocus. In contrast to methodologies that rely exclusively on sharpness assessment as a model’s labels or inputs, our approach involved the development of a targeted reward function, which has proven to markedly enhance the performance in microscope autofocus tasks. We explored various action group design methods and improved the microscope autofocus speed to an average of 3.15 time steps. Additionally, parallel “state” dataset lists with random sampling training are proposed which enhances the model’s adaptability to unknown samples, thereby improving its generalization capability. The experimental results demonstrate that the proposed liquid lens microscope with DRLAF exhibits high robustness, achieving a 79% increase in speed compared to traditional search algorithms, a 97.2% success rate, and enhanced generalization compared to other deep learning methods.

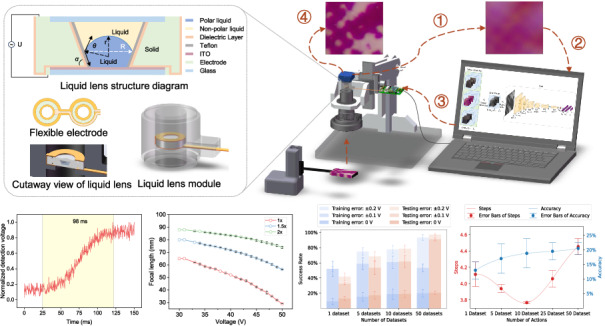

## Introduction

Microscopic observation serves as a crucial instrument in scientific research, biomedical studies, and engineering applications, ‘and has been extensively utilized across various domains, including medicine, materials science, and industry. However, traditional microscopes, characterized by intricate optical systems and focusing mechanical structures, are often cumbersome and susceptible to damage, thereby imposing significant constraints on scientific operations and application scenarios. Microscopic imaging necessitates the precise capture of samples with intricate details across an extensive range, rendering the continuous maintenance of optimal focus critically important^1^. Consequently, the miniaturization of microscopic imaging systems and the development of rapid autofocusing techniques have been longstanding objectives in relevant fields, aimed at addressing the continually evolving demands of scientific and technological advancement.

The construction of traditional microscopes typically incorporates a combination of multiple fixed-focus lenses and mechanical structures, which are employed to achieve imaging functions such as magnification and focusing. Additionally, they necessitate an adequate optical path length to enable the requisite mechanical movement for focus adjustment. Consequently, these designs are inevitably encumbered by drawbacks including bulky volumes, sluggish focusing speeds, and difficulties in enabling rapid autofocusing or operation within confined spaces^2^. In contrast, owing to the absence of mechanical components and the ability to achieve focusing by adjusting electrical signals, liquid lenses offer advantages such as compact size, rapid response, and low manufacturing costs^3–10^. Microscopes equipped with liquid lenses do not require additional mechanical parts for focusing, which effectively reduces the overall volume and enhances the efficiency of autofocusing.

The field of microscope autofocus technology has witnessed considerable advancements over the past few decades^11–18^. The advent of artificial intelligence and new optical components in recent years has led to the emergence of novel research trends in this field. Active autofocus microscopes employ the transmission and reception of specific signals to measure the distance to the object and achieve focus^19,20^. For example, Bathe-Peters et al.^21^ achieved autofocusing of an optical microscope without any mechanical motion by combining total internal reflection infrared beam ranging with an electrically adjustable lens. Similarly, Lightley et al.^22^ constructed an autofocusing system for a dual-axis optical microscope by using a superluminescent diode to emit a laser and measure the focal position of the reflected light. These methods are highly reliable and have rapid focusing speeds. However, they necessitate specialized hardware support, high system complexity, and high cost. Furthermore, passive microscope autofocus methodologies^23–28^, which assess the extent of out-of-focus and identify the focusing position exclusively through images, have been extensively investigated due to their advantageous simplicity and cost-effectiveness. Guo et al.^29^ achieved autofocusing of a microscope by calculating the phase through the acquisition of images by two pinhole-modulated cameras. DiMeo et al.^30^ proposed an autofocus control system based on Gaussian-shaped focusing measurement curves and an adaptive hill-climbing method for an autofocus control system. However, these methods usually require multiple image acquisitions and evaluations, and the focusing speed is slow.

To enhance the speed and precision of focusing, some scholars have proposed the integration of deep learning into microscope autofocusing techniques^31–35^. Pinkard et al.^1^ proposed a novel autofocusing method based on deep learning. This method is capable of predicting the position of the focal plane directly from a single out-of-focus image, utilizing convolutional neural networks. Luo et al.^36^ further developed this concept by developing a deep learning method capable of focusing in milliseconds. In a study conducted by Lightley et al.^37^ integrated a laser emitter with a convolutional neural network (CNN). The image of the laser beam reflected from a microscope coverslip was analyzed to measure the distance between the sample and the focal plane of the objective lens, thus achieving autofocus. Nevertheless, these approaches are contingent upon the quality and quantity of the training data, which may limit its efficacy in scenarios that are not previously encountered. Furthermore, these methodologies fail to consider the sequence data embedded within the focusing process, which is challenging to fully utilize and optimize. Deep Reinforcement Learning^38–40^ is capable of learning the optimal decision-making strategy through continuous trial-and-error interaction with the environment. In addition, the system is capable of utilizing sequential information to derive a more objective strategy, which is particularly well-suited to the autofocus task. Yu et al.^41^ facilitated the model’s capacity to learn the autofocus strategy directly from the input image. They implemented end-to-end optical microscope autofocus in both virtual and real environments, utilizing a discrete action space with coarse to fine steps. Chan et al.^42^ employed deep reinforcement learning to develop a noise-tolerant reward function for phase data, thereby addressing the issue of phase detection noise in autofocusing on a camera.

While these studies have contributed to the advancement of autofocusing techniques for microscopes, several challenges remain. (1) System complexity and cost: While hardware improvements have partially addressed the issue of speed, they frequently increase system complexity and cost. The utilization of liquid lenses in optical microscopes has the potential to significantly diminish the intricacy of the system. Nevertheless, the autofocus of liquid lenses still necessitates the establishment of a focal-voltage model to adjust the electrical signal focus following the calculated out-of-focus distance^43–46^, which consequently increases the pre-calibration workload. (2) Dependence on evaluation metrics: Both deep learning-based methods and deep reinforcement learning-based methods have somewhat reduced the subjectivity of manually designed contrast-based image evaluation metrics. However, the annotation of some datasets still relies excessively on image quality evaluation values. The diversity of characteristics exhibited by microscopic observation samples results in different samples being assigned distinct sharpness ratings when focusing^47^. Consequently, a reliance on image quality ratings alone may result in the model acquiring a restricted range of knowledge. (3) Insufficient generalization ability: Deep learning-based models are dependent on training data and require a substantial quantity of diverse data to enhance network performance. However, their capacity to process unknown data is not yet fully reliable. In addition, deep reinforcement learning-based autofocus methodologies typically necessitate the utilization of continuously captured images as state inputs for intelligent systems. This results in a homogeneous nature of the training data, which in turn affects the model’s generalization ability.

To address the aforementioned issues, we proposed the implementation of an adaptive Liquid Lens Microscope System that utilizes Deep Reinforcement Learning-based Autofocus (DRLAF). By leveraging the rapid response and electrical adjustment advantages of liquid lenses, this methodology employs sequential raw images as the “state” input for the deep reinforcement learning agent, to enable the model to discern objective focusing knowledge from them. Concurrently, different voltage adjustments are regarded as executable “actions” and deep reinforcement learning is employed to optimize the focusing policy. Additionally, the model is enhanced through the utilization of random sampling from parallel “state” datasets during the training phase, thereby facilitating its ability to generalize and adapt to unknown samples. The advantages of the proposed method are as follows.

- Low-cost realization: The utilization of liquid lenses in microscopes eliminates the necessity for additional, intricate zoom structures. In addition, the integration of software algorithms and simple hardware enables end-to-end optical microscope autofocusing, reducing system complexity and cost.
- Fast Response: The combination of liquid lenses with millisecond focusing speeds and intelligent focusing algorithms enables the rapid autofocusing of optical microscopes.
- Robustness: The utilization of a random sampling training method serves to enhance the model’s capacity for generalization, enabling it to effectively respond to a range of diverse samples. By adjusting the action space, it is possible to effectively address the disparate demands for speed and accuracy in microscope autofocus.

We believe that this method could provide a novel perspective and solution for achieving more objective, rapid, and high generalization capability end-to-end liquid lens autofocusing. The proposed system, which is characterized by small size, rapid response and straightforward integration of liquid lenses, has the potential for a wide range of applications in fields such as optoelectronic reconnaissance, microscopic imaging, digital lens imaging and endoscopy. It could offer robust assistance for the automation and intelligent processing of data in pertinent fields.

## Result and discussion

### System review

**Fig. 1:**
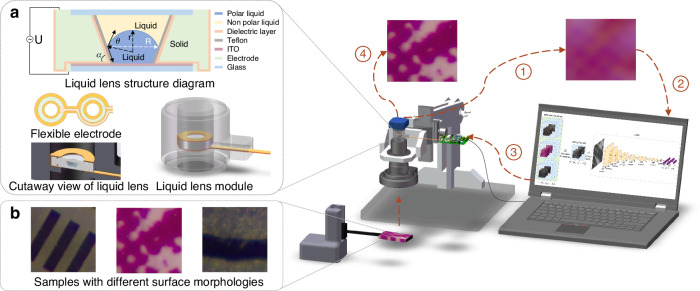
Schematic of the liquid lens microscope system utilizing deep reinforcement learning-based autofocus. **a** Structure of the EWOD liquid lens module, powered by the voltage driving board through a flexible electrode, enabling seamless integration with existing microscopes (see S1.1). **b** Training sample images with diverse surface features. The proposed random sampling method of these samples during training effectively enhances the model’s generalization capability(see Materials and Methods and S4)

Figure 1 illustrates the overall architecture of the proposed Liquid Lens Microscope System utilizing DRLAF. As shown in Fig. 1a, we constructed a microscope system equipped with a liquid lens, which operates on the principle of electrowetting for zoom control (see S1.1). The two annular electrodes of the liquid lens are located at both ends. They connect with the corresponding sections of the flexible electrodes (light green rings) to link to the host computer. The host computer runs the proposed autofocus model, based on deep reinforcement learning, which is trained using the captured image dataset. The model is capable of autonomously determining the requisite focus adjustments based on the characteristics of the current image, thereby facilitating expeditious end-to-end autofocus. Figure 1b shows the training images from different samples, including micro/nano-structured surfaces with high-contrast regular patterns, resolution test targets with medium-contrast similar patterns, and laser-processed metal substrates with medium-contrast irregular patterns. It is noteworthy that the surface features of these samples vary significantly, leading to considerable differences in image sharpness evaluation values (see S2 and S3). Such samples pose a generalization challenge for traditional deep learning methods that rely on sharpness evaluation values as labels or direct inputs. To address this challenge, this paper proposes a method using raw images as input datasets with random sampling (see Materials and Methods and S4).

### Preparation and performance of the liquid lenses microscope system

**Fig. 2:**
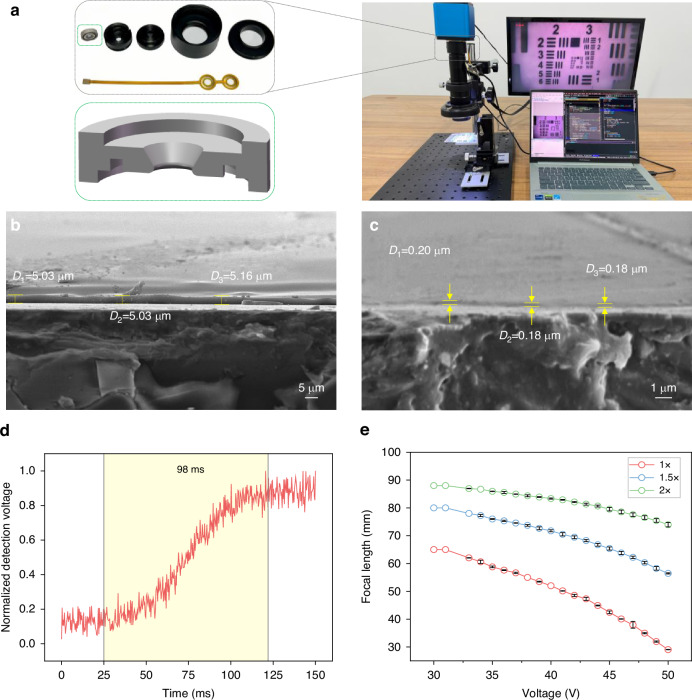
Variable-focus microscope system driven by the constructed liquid lens module and characterization of key performances. **a** Schematic of the microscope imaging system integrated with an inverted-truncated cone structured liquid lens module. **b** Scanning electron microscope image of the 5 μm thick Parylene C dielectric layer fabricated within the liquid lens. **c** Scanning electron microscope image of the 180 nm hydrophobic Teflon layer fabricated on top of the Parylene-C layer. **d** Response time curve of the liquid lens under a 40 V driving voltage. **e** Focal length-voltage variation curve of the microscope system integrated with the liquid lens module under different magnifications

The microscope was equipped with a constructed liquid lens module, which was positioned at the diaphragm location. The module was driven by a voltage source via flexible electrodes, thereby enabling the host computer to regulate the voltage. This setup formed the liquid lens microscope system, as depicted in Fig. 2a. The inner wall of the liquid lens chamber adopts an inverted truncated cone structure with a 60-degree tilt angle, a design that can improve the stability of the optical axis (see S1.2). The ports where the liquid makes contact with the floor electrodes are constructed with smoothly curved transitions. This design mitigates the concentration of electric field during the operation of the liquid lens, thereby enhancing the dielectric strength of the dielectric layer. As a result, it prevents dielectric breakdown and accelerated ageing, ultimately prolonging the lifespan of the liquid lens (see S1.3). Furthermore, the liquid lens incorporates grooves and leak-proof gaskets at its base to augment the package’s durability and compactness, hence enhancing its lifespan. Through investigating the influence of different cavity structures and dielectric materials and thicknesses on the performance of liquid lenses, a high-performance liquid lens was successfully fabricated. The lens utilizes a 5 μm thick Parylene C layer as the dielectric layer and a 180 nm Teflon AF layer as the hydrophobic coating, as illustrated in Fig. 2b and c. Figure 2d and e illustrates the response time of the constructed liquid lens and the relationship between its focal length and driving voltage under different magnification levels of the microscope, respectively. As can be observed from Fig. 2e, the focal length of the liquid lens varies at different objective lens magnifications under the same driving voltage. Therefore, for a microscope system integrated with such a liquid lens, solely relying on constructing a focal length-voltage model and performing autofocus based on this model requires substantial prior calibration work, and accuracy is difficult to guarantee. It is necessary to apply intelligent focusing algorithms in the liquid lens microscope system to achieve fast and precise end-to-end autofocus.

### Effect of actions on autofocus performance

**Fig. 3:**
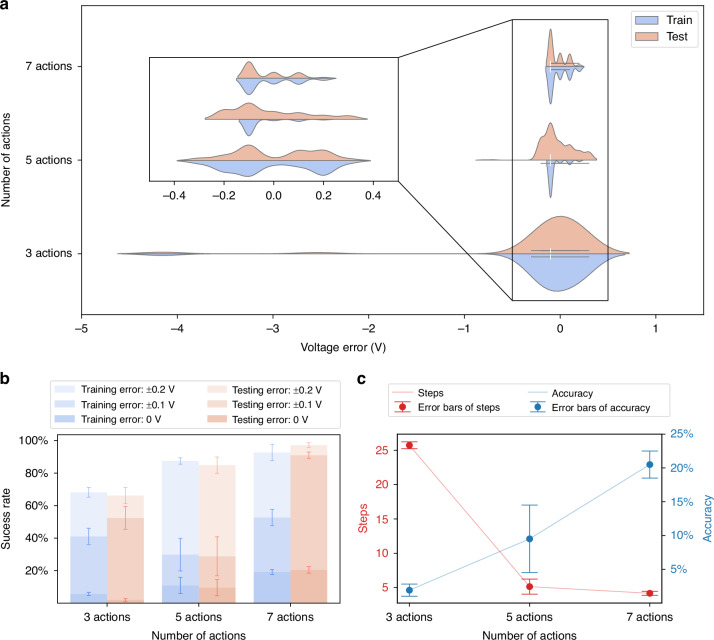
Performance of DRLAF under different action space sizes. **a** Influence of action space size on the distribution of focusing deviation in autofocusing results. As the action space size increases, the focusing voltage deviation on both the training and test sets tends to converge to 0 V. **b** Impact of action space size on the success rate and accuracy of autofocusing. As the action space size increases, both the accuracy and success rate of autofocusing significantly increase. **c** Trends of the influence of action space size on the number of autofocusing steps and accuracy. As the action space size increases, the average number of autofocusing time steps decreases significantly and stabilizes, while the average accuracy increases significantly

The autofocus adjustment actions include forward adjustments, backward adjustments, and stop actions. Moreover, the forward and backward adjustments can be divided into multiple actions depending on the size of the voltage step, collectively forming the action space. The size of the action space in DRLAF has a certain impact on the speed and accuracy of autofocusing. To determine the optimal action space size for autofocus, the performance of the model with different action space sizes was studied. Figure 3a shows the distribution of the influence of action space size on the focusing deviation of the autofocusing results. The DRLAF was trained using 50 state datasets, randomly sampled from a single sample, with the action space constructed based on a factor of 5 (see Materials and Methods for details on dataset and action processing). The results were obtained by testing the trained model 1000 times on both the training and testing datasets. The x-axis represents the deviation between the voltage selected by the model and the actual focusing voltage, while the y-axis represents the action space size. The width of each violin plot reflects the density distribution of the data. It can be observed from the figure that the size of the action space significantly affects the focusing deviation of autofocusing. As the number of actions increases, the deviation distribution tends to converge to 0 V, leading to a significant reduction in focusing deviation. At the same time, the focusing deviation on the test set also decreases significantly. This phenomenon may be attributed to the enhanced action selectivity of the model in the vicinity of the focal point position as the number of actions increases, thereby enabling more precise actions. In this study, we defined a focusing voltage deviation less than or equal to 0.2 V as successful, and a deviation of 0 V as accurate (see S6 for the definition of “successful” and “accurate”). Figure 3b shows the impact of the action space size on the success rate and accuracy of autofocusing. As the action space size increases, both the accuracy and success rate of autofocusing significantly improve. Figure 3c illustrates the influence of the action space size on the number of autofocusing time steps and accuracy on the test set. As the action space size increases, the average number of autofocusing time steps decreases significantly and stabilizes, while the average accuracy increases significantly. However, the accuracy results deviation is relatively high when the action space size is 5, which may be attributed to the trade-off between speed and accuracy. With an action space size of 5, the model has a certain degree of flexibility in action selection but may sacrifice accuracy for faster speed. On the other hand, when the action space size is 7, the model can strike a balance between speed and accuracy by selecting appropriate actions, thereby increasing the stability of autofocusing.

The findings of the preceding research indicate that an increase in the size of the action space can lead to an improvement in the performance of the model. However, the specific configuration of the action set, including step sizes and scaling ratios, also plays a crucial role. Given that an action set with 7 actions achieves better focusing performance, Table 1 compares the accuracy and time steps for different configurations of these 7 actions. Detailed methods for configuring the action sets are provided in the Materials and Methods section. From the table, we can observe the following: First, smaller bases effectively improve the autofocusing accuracy, with accuracy increasing as the base decreases within a certain range. Second, both excessively large and small action control factors, obtained by magnifying the base through the two methods, increase the number of time steps required for autofocusing. Overall, the action sets determined by the logarithmic method with smaller bases are more suitable. Taking bases 3 and 5 as examples, each has its advantages and disadvantages. A smaller base can achieve higher autofocusing accuracy, while a larger base can realize faster focusing speed. Therefore, an appropriate base can be selected based on specific requirements in actual processes.

**Table 1:** Performance of DRLAF under different action sets

Base	Method	Action control factor	Time step	Accuracy
2	Logarithm / Multiple	(-4,-2,-1,0,1,2,4)	7.2	22.3%
3	Logarithm	(-9,-3,-1,0,1,3,9)	4.387	32.2%
	Multiple	(-6,-3,-1,0,1,3,6)	5.322	23.1%
5	Logarithm	(-25,-5,-1,0,1,5,25)	3.15	20.7%
	Multiple	(-10,-5,-1,0,1,5,10)	4.454	20.5%
7	Logarithm	(-14,-7,-1,0,1,7,14)	5.544	14.4%
	Multiple	(-49,-7,-1,0,1,7,49)	3.739	14.6%

### Effect of state random sampling in training on autofocus performance

**Fig. 4:**
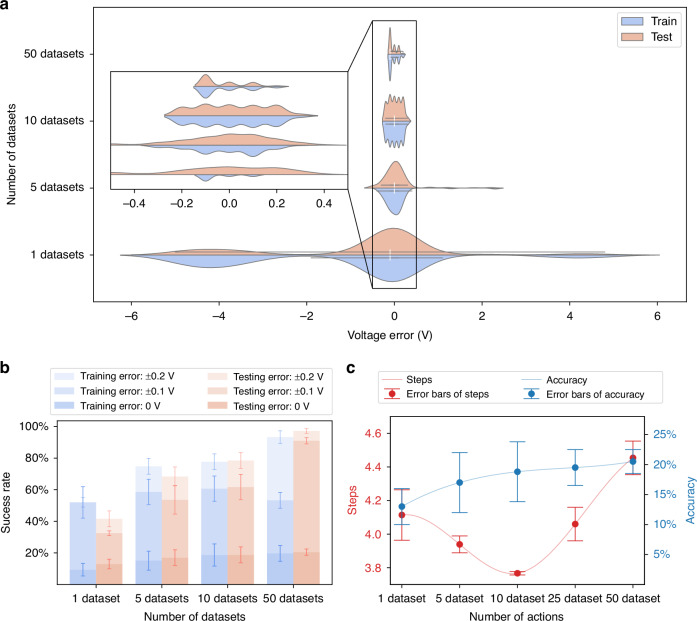
Influence of state dataset list size on DRLAF trained by randomly sampling performance. **a** Impact of state dataset list size on the distribution of automatic focusing deviation. As the number of state datasets increases, the focusing deviation of the model significantly decreases. **b** Effect of state dataset list size on automatic focusing success rate. With an increase in the number of state datasets, both the accuracy and success rate of automatic focusing notably improve. **c** Influence of state dataset list size on automatic focusing time steps and accuracy. The trend shows that as the number of state datasets increases, the accuracy of automatic focusing gradually improves, but the average step exhibits a trend of initially decreasing and then increasing. Appropriate sizes of state dataset lists can be configured based on practical requirements

The proposed method introduces a deep reinforcement learning approach trained by random sampling from multiple state datasets (see Materials and Methods). This approach is aimed at enhancing the model’s autofocusing success rate and improving its generalization across diverse samples. Specifically, samples are grouped into multiple sets of state datasets based on various perspectives. These sets are then aggregated into a single category as a state dataset list, from which training is conducted by randomly sampling list elements. See Materials and Methods for specific treatment of training and test datasets. The results were obtained by testing the trained model 1000 times on both the training and testing datasets. Figure 4 illustrates the impact of state dataset list size on the model’s autofocusing performance. Figure 4a shows the influence of state dataset list size on the distribution of focusing deviations. The x-axis represents the deviation between the voltage selected by the model and the actual focusing voltage, while the y-axis represents the number of state datasets for random sampling. It is observed that with an increase in the number of state datasets, the focusing deviation significantly decreases, trending converge to 0 V. Figure 4b presents the effect of state dataset list size on autofocusing success rate and accuracy. As the number of state datasets increases, both the accuracy and success rate of autofocusing notably improve. Additionally, the model’s performance on the test sets gradually surpasses that on the training sets. With 50 datasets, the model achieves a 97.2% success rate on the test set, along with a root mean square error (RMSE) of $2.85\times {10}^{-3}$ V for the predicted voltage, indicating an improved ability to generalize. This trend may be attributed to the model learning more comprehensive and diverse information as the number of state datasets increases. This approach reduces reliance on individual sample states, mitigates the impact of noise on model performance, and lowers the risk of overfitting. As a result, the model is better able to comprehend and adapt to various autofocus tasks. Figure 4c demonstrates the trend of state dataset list size on the number of autofocusing time steps and accuracy on the test set. As the number of state datasets increases, the accuracy of autofocusing shows a gradual improvement. However, the average time steps demonstrate a trend of initially decreasing and then increasing. This phenomenon can be attributed to the model’s ability to learn more effective feature representations from increasingly rich and diverse datasets, which enhances its generalization to previously untrained data. Nevertheless, an expansion in the dimensions of the dataset list entails an augmentation in the prevalence of noise and randomness, thereby prompting the model to favor robust actions, which in turn elevates the mean number of time steps. Therefore, in practical applications, appropriate sizes of state dataset lists can be configured based on the task type and varying requirements for precision and speed.

### Generalization experiments

To further evaluate the model’s generalization capability on unknown samples, we conducted specific autofocusing experiments. Three methods are tested on completely unfamiliar sample datasets. Method 1: Deep Learning-based Automatic Focusing Model^35^. Method 2: DRLAF trained by random sampling from a single sample. Method 3: DRLAF trained by random sampling from different samples. The result is shown in Table 2. Since deep learning and deep reinforcement learning have different requirements for datasets, the training data scale for Method 1 remains constant. In Method 3, two different types of samples are utilized for training (see Materials and Methods). Compared to Method 2 and Method 1, Method 3 demonstrates significantly higher autofocusing success rates on unfamiliar datasets. This result further validates the effectiveness of the random sampling training strategy in enhancing model generalization. By diversifying the state dataset and reducing model overfitting, this approach improves the model’s ability to adapt to variations across different samples. Furthermore, it was observed that the success rate of the automatic focusing process on datasets that were not previously encountered improved as the number of state datasets used for training increased. This indicates that broadening dataset coverage horizontally and expanding dataset scale vertically holds promise for further enhancing model generalization. It is noteworthy that even with the minimal training data volume, Method 3 outperforms the other two methods, demonstrating its applicability in extending existing data.

**Table 2:** The autofocus success rate of different models for completely unfamiliar samples

Dataset list size	Method 1	Method 2	Method 3
2	0%	0%	5.4%
10	0%	0%	5.6%
20	0%	1.5%	12.9%

### Ablation experiments on the reward function

**Fig. 5:**
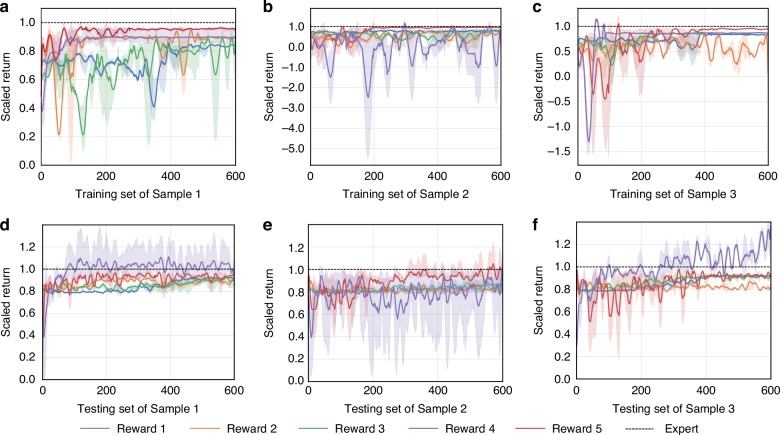
Mean and standard deviation of scaled returns for the DRLAF trained by random sampling using 5 different reward functions and 3 random seeds on the training and testing sets of 3 different samples

**Fig. 6:**
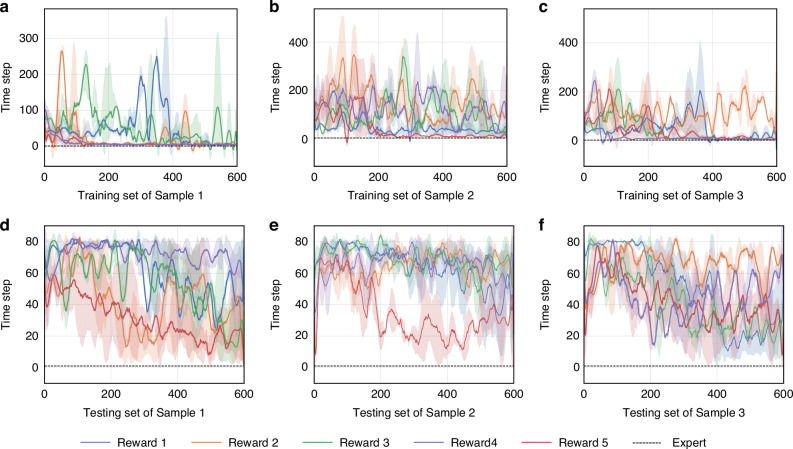
Mean and standard deviation of timesteps for the DRLAF trained by random sampling using 5 different reward functions and 3 random seeds during the training process on the training and testing sets of 3 different samples

The present study proposes a hybrid reward function design (see Methods and Materials) that enhances model performance, particularly in autofocus tasks. This is achieved by incorporating stop, time step, and additional reward components into the sharpness evaluation. To thoroughly analyze the impact of the proposed hybrid reward function design and the contribution of each reward component to the algorithm’s performance, a series of ablation experiments were conducted. Specifically, the action space was configured with a set of 7 actions based on a base of 5, and DRLAF was trained by random sampling. The reward function variations are as follows: Reward 1: Sharpness Reward, Reward 2: Sharpness + Stop Reward, Reward 3: Sharpness + Time Step Reward, Reward 4: Sharpness + Additional Reward, Reward 5(the proposed reward): Sharpness + Time Step + Stop + Additional Reward (Table 3). Figure 5 presents the results of the ablation experiments on the reward function, showing the scaled return during the training process on different samples for both the training and testing sets. Figure 6 displays the time step results during the training process on different samples for both the training and testing sets. The figures illustrate that during training, Reward 5 exhibits the best convergence across all three samples. For Reward 4, the significant increase and substantial fluctuations in the return on the test set, along with the time step performance on the test set presented in Fig. 6, suggest that the model cannot terminate autonomously. Consequently, its performance on the test set is characterized by oscillatory behavior, leading to anomalous return values. The results presented in the figures indicate that Reward 5 is the most effective reward function formulation investigated, as it exhibits consistent convergence across different samples while achieving a significant reduction in the number of time steps required.

**Table 3:** Ablation results of reward function

Number	Reward Function Variant	Average Score	MAE	Time Steps	Focusing Accuracy	Success Rate
1	Sharpness Reward	735.01	0.94	∞	0%	0%
2	Sharpness + Stop Reward	-436.19	13.06	3.55	10%	54%
3	Sharpness + Time step Reward	-216.88	4.65	3.5	12%	55%
4	Sharpness + Additional Reward	1259.87	1.37	∞	13%	0%
5	Sharpness + Time step + Stop + Additional Reward	35.4	1.43	3.15	16%	83%

### Autofocus experiment

**Fig. 7:**
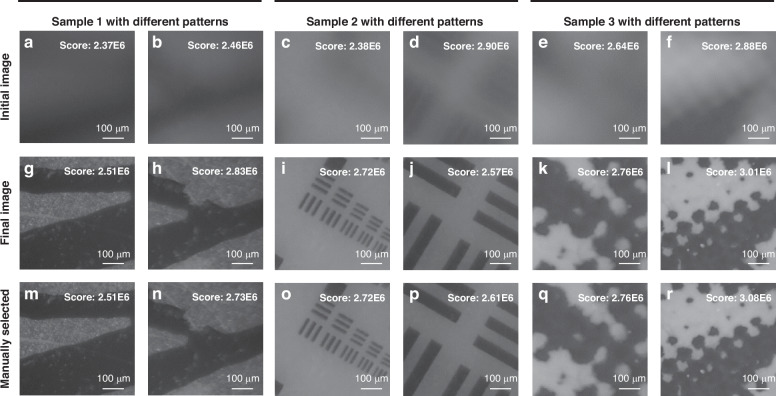
Experimental results of DRLAF. **a**–**f** Initial images with random defocus amounts, exhibiting lower clarity evaluation values and noticeable differences among different samples. **g**–**l** Images obtained after autofocus demonstrate significantly improved clarity evaluation values. **m-r** Manually selected focused images, indicating that the results obtained from autofocus are comparable to those obtained manually, both in visual observation and clarity evaluation metric values

Figure 7. illustrates the imaging performance of the proposed autofocus algorithm under different samples and random initial defocus states. Figure 7a–f depict scenes with random defocus amounts, exhibiting severe loss of details and lower clarity evaluation values. Following autofocusing by the proposed method, the resulting images (Fig. 7g–l) demonstrate rapid adjustment to achieve well-focused images regardless of the initial defocus severity, with clear details and significantly improved corresponding evaluation metric values. It is noteworthy that due to inherent differences in surface features among different samples, there are noticeable variations in clarity evaluation values between focused and defocused states. Whether comparing different fields of view within the same sample or across different samples, sharpness differences are minor in defocused states but become pronounced when in focus (e.g., Group 1 (a, g) vs. Group 2 (b, h), and Group 1 (a, g) vs. Group 3 (d, j)). This indicates that solely relying on clarity evaluation values is challenging for accurately determining the optimal focus state. To assess algorithm performance, we compare the autofocus results with manually focused results (Fig. 7m–r). Although distinguishing between the two is difficult when observed directly, the precision of the algorithmic output images is comparable to, and in some instances exceeds, the level of manual focusing, as indicated by evaluation metrics. The experimental findings indicate that the proposed autofocus method can rapidly and effectively adjust imaging systems from any initial state to the focused position. This results in high-quality images that are unaffected by sample differences and are comparable to the results of manual focusing.

To provide a more comprehensive assessment of the proposed autofocus method, two additional approaches, namely the golden section search-based^48^ and the Fibonacci search-based^49^ autofocus methods, are implemented in the system. The value obtained from the Energy clarity evaluation function shown in Equation S5 is used as the evaluation index. The experiments were conducted on three distinct samples of each of the 10 viewing positions, commencing with an arbitrary amount of defocus for autofocusing. The resulting data for the average autofocusing time step are presented in Table 4. Experimental results demonstrate that the proposed model achieves autofocus in an average of only 3.15 steps, reducing the time by 79% compared to the golden section search-based method and by 60.63% compared to Fibonacci search-based methods.

**Table 4:** Time steps for different autofocus methods‘

Sample	Golden Section	Fibonacci	Ours
1	15	8	3.14
2	15	8	3.16
3	15	8	3.15

## Conclusions

This study proposes an innovative liquid lens microscope system that achieves rapid and precise autofocus by utilizing deep reinforcement learning. Firstly, a liquid lens driven by the electrical dielectric wetting principle was fabricated, offering the advantages of small volume and fast response speed, which can effectively improve the structural compactness and zoom speed of microscopes when integrated. Secondly, an end-to-end autofocus is achieved by training a deep reinforcement learning model, further enhancing the focusing speed. Concurrently, a reward function tailored for the autofocus task was designed, enabling the model to focus more rapidly and autonomously. Furthermore, several action group design methods were introduced, which effectively enhance the speed and accuracy of autofocus by adjusting key parameters. In the experiments, an average of 3.15 steps was required to achieve autofocus, representing a 79% and 60.63% improvement in speed compared to traditional search algorithms. Additionally, a novel method for random sampling from multiple “state” dataset lists was proposed to address the limitation of model sensitivity to only the trained data. By increasing the number of state datasets, the model’s ability to extract common features was significantly enhanced, enabling reliable autofocus across different samples and fields of view. With the expansion of the state datasets to 50, the model achieved a 97.2% success rate, with a root mean square error (RMSE) of $2.85\times {10}^{-3}$ V for the predicted voltage on the test set. This result demonstrates that the trained agent developed a robust autofocus strategy that is not dependent on the training data, thereby improving its generalization capability. The proposed liquid lens microscope system utilizing DRLAF significantly simplifies the structural complexity, enhances system compactness, reduces operational difficulty, and increases focusing speed. It has broad application prospects in fields such as electro-optical reconnaissance, microscopic imaging, digital lens imaging, and endoscopy, providing robust support for automation and intelligence processes in related fields.

## Materials and methods

### Liquid lens

We implemented adjustable focusing functionality using a custom-made liquid lens based on the electrowetting-on-dielectric (EWOD) effect. As depicted in Fig. 1a, the liquid lens consists of an upper glass cover plate, a lower ITO cover plate, a chamber, a Parylene C dielectric film coated on the chamber, and a hydrophobic Teflon AF film. The Parylene C film, with a thickness of 5 μm, was prepared using chemical vapor deposition, while the 180 nm-thick Teflon film was fabricated via spin-coating. It is noteworthy that we designed the cavity with a conical structure featuring rounded corners. On one hand, this structure aids in stabilizing the optical axis centre and reducing image distortion. On the other hand, it also facilitates the uniformity of the dielectric Parylene C film and the hydrophobic PTFE membrane on the inner walls of the cavity, thereby preventing membrane damage and improving device yield. Specific dimensional parameters of the liquid lens are listed in Table S1.

### Dataset processing

In this work, we utilize the image sequences collected during the autofocus process of the liquid lens as the state input for the reinforcement learning agent, aiming to provide it with sufficient visual information to guide the learning of the autofocus strategy. The experimental methodology employs a microscope with a fixed object distance, equipped with a voltage-driven liquid lens. The voltage is adjusted in increments of 0.1 V, allowing for changes in focal length to capture the complete focusing process (defocused-focused-defocused) of the sample. After processing the images, a batch suitable for input into the DRLAF is obtained, forming a “state” dataset denoted as S. S is a four-dimensional tensor with dimensions 100 × 1 × 224 × 224, representing 100 frames of single-channel grayscale images with a resolution of 224 × 224 pixels. The above steps are repeated for the same sample at different fields of view, along with data augmentation, resulting in 100 state datasets for the same sample. This process is also repeated for different samples, generating 100 state datasets for each. The training and testing datasets are derived from these datasets but do not overlap.

The study proposes a random sampling training method, where multiple state datasets are combined into a list L for training (as shown in Fig. S7). During each iteration, a state dataset S_i_ is randomly selected from L. The testing set, while equal in length to the training set, contains distinct state datasets. Figure 3 focuses on the effect of actions on autofocus performance, utilizing 50 state datasets of the same sample for training and another 50 for testing. Figure 4 investigates the impact of different numbers of randomly sampled training datasets on performance, with training datasets comprising 1, 5, 10, and 50 state datasets, and testing datasets of corresponding sizes. For Method 3 in Table 2, two different types of samples are utilized for training. The number of state datasets for each sample is set to half of the total list size. Additionally, experiments are conducted using extra unfamiliar samples. As the training data encompasses observation targets with various features, the reinforcement learning model can learn multiple focusing states simultaneously, acquiring more universal knowledge and thereby enhancing its generalization capability.

### Reward function

The reward function plays a crucial role in the reinforcement learning framework by defining task objectives, evaluating actions, shaping policies, and providing feedback. Therefore, for the autofocus task, it is necessary to consider multiple factors such as image sharpness, focusing time, and stopping strategy to provide clear behavioral feedback and learning goals for the agent. As a result, we design the following reward function:

1 $$r=-\alpha \ast \frac{{F}_{c}-{F}_{min}}{{F}_{max}-{F}_{min}}-\beta \ast log\frac{n}{\beta }+\mu \ast Done+\delta$$

where, image sharpness is the core indicator of the autofocus task, and the first term of the reward function is constructed based on image sharpness. Various sharpness evaluation functions were assessed (see S5), and Equation S6 was adopted to quantify the sharpness of images captured by adjusting the liquid lens. The evaluation values Fc are normalized to ensure consistency across different images taken under varying voltages. The parameter α acts as a scaling factor that maps these values to a negative range $[-\alpha ,0]$, thereby enhancing the agent’s sensitivity to variations in image clarity and improving its efficiency in searching for the optimal focus. Fast-focusing is another important objective of the task, hence the second term $\beta \ast log\frac{n}{\beta }$ represents the time step reward component, where n is the current focusing execution step and $\beta$ is the step penalty coefficient. The logarithmic nature of the function ensures a gradual decrease in the reward as the number of focus adjustment steps increases. When the step count falls within the range $(0,\beta )$, the reward remains positive. However, once the step count surpasses *β*, the reward becomes negative. This design encourages the agent to minimize the number of steps required to achieve the desired focus, promoting efficiency in the focusing process. The third term $\mu \ast Done$ is the stopping reward component, μ represents the reward coefficient and $Done$ is a Boolean flag. The agent receives the $\mu$ reward only when it correctly stops near the optimal focus point, signaled by $Done=1$. This term ensures that the agent halts its adjustments at the target focus, avoiding the excessive adjustments that could inflate the reward. The last term $\delta$ is an additional reward component aimed at enhancing the discriminative ability of the reward function by setting relatively large positive and negative rewards for the clearest and least clear images, respectively, thereby further reducing the focusing steps. Since achieving clear imaging, reducing the time to focus, and stopping automatically are all equally important in the autofocus task, the maximum absolute values of each term should be on the same order of magnitude. This prevents the agent from becoming overly biased toward a single term, ensuring it can complete the overall objective effectively. In this study, the final parameter values are: $\alpha =100$, $\beta =30$, $\mu =200$, and $\delta =100$.

### Action space

In the proposed reinforcement learning autofocus model, we design the agent’s executable actions to adjust the voltage applied to the liquid lens with different step sizes. Specifically, the magnitude of action (a) is determined by the following equation:

2 $$a=\tau \times v$$

where $\tau$ is the step size scaling coefficient, and $v$ is the resolution of the voltage adjustment.

Firstly, assuming the state space $S=\{{s}_{1},\,{s}_{2},\ldots ,{s}_{i},\ldots ,{s}_{n}\}$ with a cardinality of $\Vert S\Vert ={n}_{S}$, the action space $A=\{{a}_{1},{a}_{2},\ldots ,{a}_{j},\ldots ,{a}_{n}\}$ with a cardinality of $\Vert A\Vert ={n}_{A}$, where ${n}_{A}\ge 3$ (including three basic actions: forward, backward, and stop). To ensure that ${n}_{A}$ satisfies certain mathematical constraints, we leverage the properties of the logarithmic function, allowing mapping the variations of ${n}_{A}$ and $\tau$ to a small range of variations in the base $b$ of the logarithmic function. Specifically:

3 $$\frac{{n}_{a}-1}{2}\,<\,lo{g}_{b}\frac{{n}_{s}}{2}$$

where $b$ is a parameter controlling the transformation of the action space, thus the step size scaling coefficient $\tau ={{b}^{j}}$.

In the experiments investigating the impact of action space size on autofocus performance, we adopted the method described by Eq. (3), setting $b=5$ to determine different values of ${n}_{A}$ and their corresponding action sets. Meanwhile, in experiments investigating the influence of action set composition on performance, the same approach was followed by selecting prime numbers less than 10 ($\{2,3,5,7\}$) for $b$ to obtain corresponding ranges of ${n}_{A}$ values and constructing complete action sets within each range. Additionally, action sets were constructed for comparative analysis by scaling the minimum step size coefficient ${\tau }_{0}$ by multiples of a common base $b$($\{{\tau }_{0}=b,{\tau }_{1}=2b,{\tau }_{2}=3b\}$) using the same base $b$ values $\{2,3,5,7\}$.

## Supplementary information

Autofocus video of the liquid lens microscope system using DRLAF

Supplemental Material

Data Set 1

Data Set 2
