# Supplementary material for: Precision autofocus in optical microscopy with liquid lenses controlled by deep reinforcement learning
Source: Microsyst Nanoeng. 2024 Dec 24;10:201. doi: 10.1038/s41378-024-00845-8 (PMC11668857; doi:10.1038/s41378-024-00845-8)
Supplement: Supplementary file 2 — Supplemental Material [file 41378_2024_845_MOESM2_ESM.docx]

SUPPLEMENTARY INFORMATION

Precision Autofocus in Optical Microscopy with Liquid Lenses Controlled by Deep Reinforcement Learning

Jing Zhang^1^, Yong-feng Fu^2^, Hao Shen^1^, Quan Liu^2^,

Li-ning Sun^1^, and Li-guo Chen^1,*^

^1^ *School of Mechanical and Electrical Engineering, Soochow University, No.8 Jixue Road, Suzhou City, Jiangsu Province, 215000, China.*

^2^ *School of Computer Science and Technology, Soochow University, No.333 Ganjiang East Road, Suzhou City, Jiangsu Province, 215006, China.*

**Correspondence: LG Chen, E-mail:* [*chenliguo@suda.edu.cn*](mailto:chenliguo@suda.edu.cn)

12 pages, 7 figures, 1video: S1-S5

This document provides supplementary information to "Precision Autofocus in Optical Microscopy with Liquid Lenses Controlled by Deep Reinforcement Learning". It includes the principles of the liquid lens, formula derivations, and experimental studies on the fabrication process, along with the detailed model framework. Additionally, it covers the differences in image sharpness across various samples and the corresponding sharpness evaluation functions.

S1: Details of the electrowetting-based liquid lens

S1.1: Mechanism research

Liquid lens zooming primarily relies on the dielectric wetting principle, as shown in Fig. S1. When a voltage is applied between a polar droplet and an electric conductor it contacts, the wettability of the liquid on the surface of the conductor increases. This leads to a shift of the three-phase contact line (solid-liquid-gas), causing the droplet to spread along the solid surface[1]. The variation of the contact angle of a droplet on a solid surface with voltage can be determined from the Young-Lippmann equation:

$$\cos\theta_{U}=cos\theta_{0}+\frac{\varepsilon_{0}\varepsilon U^{2}}{2d\sigma_{LY}}$$

Fig. S1 Principle of EWOD.

where $\theta_{U}$ is the contact angle after an externally applied voltage (U), $\theta_{0}$ is the contact angle when the voltage is 0 V, $\varepsilon_{0}$ is the vacuum dielectric constant, $\varepsilon$ is the dielectric constant, $\sigma_{LY}$ is the surface tension between the liquid and the gas, and d is the thickness of the dielectric layer.

By injecting a polar liquid into a three-dimensional capillary cavity fabricated from a conductive solid based on the electrowetting principle, the three-phase contact line is allowed to transition its motion from planar to perpendicular direction, consequently increasing the range of liquid surface curvature variation, as shown in Fig. 1a. Within this polar liquid, introducing an immiscible and density-matched non-polar liquid reduces the liquid-liquid interfacial tension and increases the initial contact angle, therefore further amplifying the displacement of the three-phase contact line. When voltage is applied, as the three-phase contact line moves along the capillary wall while maintaining a constant liquid volume, deformation of the two liquid interfaces occurs. Due to the refractive index mismatch between the two liquids, this deformation of the liquid interface curvature leads to a variation in the focal length of the liquid lens, thereby enabling the zoom function.

In a liquid lens in the shape of a truncated cone, the radius $R$ of the three-phase contact line is related to the contact angle $\theta$ by $\cos(\theta-\alpha)=\frac{R}{r}$ Based on the simplified lens maker's formula $\frac{1}{f}=\frac{N-1}{r}$ for the liquid lens model, the formula for the focal length of a liquid lens can be derived as formula S2.

$$f=\frac{R}{(N-1)\cos(\theta-\alpha)}$$

Where $R$ is the equivalent capillary radius, i.e., the radius of the three-phase contact line, $r$ is the radius of curvature of the polar liquid surface, $f$ is the focal length of the lens, and $N$ is the refractive index of the liquid. It should be noted that as the voltage driving the liquid lens changes, the radius R will vary with the height of the three-phase contact line. In addition, the radius R varies with the polar liquid volume and the driving voltage. Hence, the polar solution volume is also a factor affecting the liquid lens performance.

S1.2: Effect of dielectric material and thickness on the properties of the liquid lens

Fig. S2 Liquid lens response time for different cavity minor diameters (d) and inclination angles (α).

Decreasing the inclination angle α of the liquid lens cavity can increase the initial contact angle and improve optical axis stability [2]. However, as the tilt angle decreases, the liquid volume increases significantly, which will affect the lens performance. On the other hand, the sharp edge of the bottom aperture reduces the strength of the dielectric layer. By adopting a fillet transition design, the cavity aperture diameter can be expanded from 3mm to 4mm, while effectively increasing the breakdown voltage of the liquid lens.

Fig. S2 shows the response time of liquid lenses with different cavity minor diameters ($d$) and tilt angles ($\alpha$). Among them, the 3mm-60° and 3mm-45° cavities without fillet transition fail after the voltage exceeds 80 V, while the 4mm-60° liquid lens can operate stably at 100 V.

**S1.3: Effect of dielectric material and thickness on the properties of liquid lens**

The performance of electrowetting-based liquid lenses can be influenced by the dielectric constant of the insulating material and the thickness of the dielectric film. Figure S3 illustrates the experimental studies carried out on the dielectric wettability of different dielectric materials. We employ an ITO glass plate as a substrate and coat different dielectric films of about 5 μm thickness on its surface, respectively, and then cover a hydrophobic layer of polytetrafluoroethylene (Teflon) of about 180 nm. By adjusting the voltage between the droplet contact electrode and the ITO electrode on the substrate, we measured the voltage-dependent contact angle changes of the droplets (as shown in Figures S2c and S2d).

**Fig. S3 Electrowetting performance study of different dielectric films. a** Effect of different dielectric materials on contact angle and driving voltage. **b** Driving voltage and threshold voltage of parylene C at different thicknesses. **c** Initial contact angle of the droplet. **d** Contact angle of the droplet after applying voltage.

The experimental results revealed that parylene C exhibited a significant low-voltage driving advantage and a considerable contact angle variation range at the same thickness, as illustrated in Fig. S3a. Fig. S3b depicts the trends of driving voltage and threshold voltage as a function of parylene C film thickness. Ultimately, we determined the optimal dielectric layer parameters: a 5 μm thick parylene C film covered with a 180 nm Teflon hydrophobic layer (as shown in Fig. 2b, c), which effectively enhances surface hydrophobicity and minimizes contact angle hysteresis.

S1.4: Parameters for liquid lens preparations

Based on the above study, we fabricated the liquid lens for the EWOD principle with the specific parameters shown in Table S1.

Table S1 Parameters for liquid lens preparations.

| **Parameter** | **Preparation Technics** | **Standard** |
| --- | --- | --- |
| ITO Glass | Magnetron sputtering | 7-10 Ω |
| Parylene C | Chemical Vapour Deposition (CVD) | 5 μm |
| Teflon | Spin coating | 180 nm |
| Cavity minor diameters（d） | Precision machining | 4 mm |
| Cavity inclination angles (α) | Precision machining | 60° |
| Cavity height（H） | Precision machining | 3 mm |

S2: Variability in clarity across samples

Existing image clarity evaluation methods based on the airspace domain mainly use the pixel grey scale difference to obtain the value through the clarity evaluation function to distinguish the sharpness of different images. Traditional autofocus methods then guide the focusing process based on these evaluated values. However, due to variations in sample surface topography, the values obtained from pixel grayscale-based clarity evaluation functions also exhibit differences. Fig. S4. shows the measured clarity changes of different samples during the focusing process. It can be observed that the clarity variation trends and peak-focused clarity values differ across different samples. Furthermore, Fig. S5. demonstrates that even for the same sample, the focused clarity can fluctuate to varying degrees depending on the observation location. Consequently, traditional autofocus methods based on image clarity variation trends, as well as deep learning approaches relying solely on single-image clarity values, may be limited in their generalization capabilities across different samples.

Fig. S4 Variation of sharpness with object distance for samples with different surface morphologies and patterns.

Fig. S5 Variation of focus sharpness with viewing position for different samples.

S3: Clarity evaluation function

In the null domain, the focused image more defined edges and finer features compared to the defocus image. The sharpness of the image can be assessed by employing the clarity evaluation function based on the null domain. Image clarity evaluation functions based on the null domain include the Energy of Gradient function, Laplace function, Brenner function, Tenengrad function, SMD function, etc. Nevertheless, the clarity evaluation functions yield varying assessment values for distinct images in terms of clarity differentiation. This study selects four representative functions to assess and compare the samples.

(1) Brenner function [3]:

The Brenner gradient function is a basic method for evaluating gradients. It computes the squared difference between the grey levels of two neighbouring pixels. The formula for this function is as follows:

$$F_{Brenner}=\sum_{\text{x}}^{X}\sum_{\text{x}}^{Y}[I(x+2,y)-I(x,y)]^{2}$$

where $I\left(x,y\right)$ denotes the grey value of the pixel point at the position $\left(x,y\right)$.

(2) SMD function [4]:

The SMD function quantifies the image sharpness by calculating the difference between the grey values of neighbouring pixels in the horizontal and vertical axes and subsequently summing them. The function is defined as follows:

$$F_{SMD}=\sum_{\text{x}}^{X}\sum_{\text{x}}^{Y}|I(x+1,y)-I(x,y)|+|I(x,y+1)-I(x,y)|$$

(3) Energy of Gradient function(EOG) [5]:

The technique utilizing the variation in pixel grey values is proficient in extracting edge information, but it is highly susceptible to noise. The EOG function is an improved method based on the SMD function, the absolute value of the grey difference is changed to the sum of squares, which can increase the magnitude of the gradient, and it can filter the noise of the low gradient variations. The EOG function is defined as follows:

$$F_{EOG}=\sum_{\text{x}}^{X}\sum_{\text{x}}^{Y}[I(x+1,y)-I(x,y)]^{2}+[I(x,y+1)-I(x,y)]^{2}$$

(4) Laplaciant function [6]:

The Laplacian function mainly sums the squares of the pixel grey levels of the image after doing the convolution operation with the Laplacian operator as the clarity evaluation value of the image, where the Laplacian operator is illustrated in Eqn. S6 and the Laplacian function is illustrated by Eqn. S7:

$$L=\frac{1}{6}\left(\begin{matrix} 0 & -1 & 0 \\ -1 & 4 & -1 \\ 0 & -1 & 0 \end{matrix}\right)$$

$$F_{Laplacian}=\sum_{\text{x}}^{X}\sum_{\text{x}}^{Y}[G(x,y)]^{2}$$

where $G\left(x,y\right)$ is the result of convolving the image with the Laplacian operator.

This paper utilizes the aforementioned functions to distinguish between the focusing process images. The outcomes are presented in Fig. S6., which reveals that the Energy function exhibits the greatest ability to differentiate between images with varying degrees of blurriness. Consequently, this paper employs the value of the Energy function as an indicator for image evaluation within the reward function.

Fig. S6 Discrimination of focusing process images by different clarity evaluation functions.

S4: Model training

The proposed model consists of two deep neural networks: the policy network (parameter is $\theta$) and the target network (parameter is $\hat{\theta}$ $)$. During the training process, the agent selects an action $a_{i}$ based on the current state $s_{i}$ using an ϵ-greedy policy. The agent then executes action $a_{i}$ to transition to the next state $s_{i+1}$. This process is repeated, and the networks are updated until the voltage is adjusted to the vicinity of the clearest image position. Then, the next iteration of training begins.

**Fig. S7 DRLAF Model Framework. a** Training Procedure of DRLAF. **b** Architecture of DRLAF Network**.**

The specific training process is illustrated in Fig. S7a. At initialization, both the policy network $Q(s,a;\theta)$ and the target network $\hat{Q}(s,a;\hat{\theta})$ have the same network architecture (as shown in Fig. 8b) and parameters $\theta=\hat{\theta}$. At the beginning of each iteration, an initial state $s_{1}$ is obtained. During the focusing process, based on the current state $s_{i}$, an action $a_{i}$ is selected either randomly with decay probability or by choosing the action a_i_ with the maximum Q-value, $a_{i}=max(Q\left(s_{i},a;\theta\right))$. The selected action $a_{i}$ is executed to transition to the next state $s_{i+1}$, resulting in an immediate reward $r_{i}$ and two boolean values: *isAchieved* (indicating whether the vicinity of the optimal focus position is reached) and *Done* (which is true only when isAchieved=True and a_i_ is a stop action). The tuple ($s_{i},a_{i},r_{i},s_{i+1},Done$) is then stored in the experience pool $D$.

When the amount of data in $D$ is sufficient, batch data is randomly sampled from it. The target Q-value $y_{i}$ is computed using the target network, given by $y_{i}=r_{i}+\gamma\max(\hat{Q}(s_{i+1},a';\hat{\theta})$, where $\gamma$ is the reward decay factor. The loss is calculated using the policy network as $Loss=\frac{1}{N}\sum_{i=1}^{N} (y_{i}-Q\left(s_{i},a_{i};\theta\right))^{2}$and the policy network is updated by minimizing the loss. The target network is updated with the policy network for every fixed number of actions.

S5 Definition of Successful and Accurate Focusing

In this paper, two critical terms related to autofocus performance are introduced: "accurate" and "successful" focusing. This section provides a detailed explanation of these definitions and their foundation.

**Justification**

As illustrated in Fig. S8, images captured near the focal point exhibit minimal perceptual differences despite slight variations in focusing voltage. This observation indicates that defining a singular optimal solution may not be the most efficient approach for practical autofocus systems. Instead, focusing performance can be better characterized by a range of acceptable solutions, where image clarity remains sufficiently high.

**Fig. S8 Images near the focal point which NS is the normalized sharpness score.**

To further validate this hypothesis, a series of experiments was conducted utilizing three different samples, with a voltage adjustment resolution of 0.1 V. The normalized sharpness curves derived from these experiments, as depicted in Fig. S9, demonstrate that the overall trends in image sharpness are consistent across different samples. The normalized sharpness values near the focal point for each sample are summarized in Table S2. Based on these trends, a standardized criterion based on normalized sharpness can be applied across different samples, thereby ensuring consistency in autofocus performance.

**Definition of Accurate Focusing**

Accurate focusing is defined as the position at which the normalized sharpness reaches 1, corresponding to a voltage deviation of 0 V. At this position, image clarity is maximized for all three samples. This definition serves as the benchmark for identifying the optimal point of focus within the autofocus system.

**Definition of Successful Focusing**

The experimental data presented in Table S2 indicates that the normalized sharpness remains above 0.8 for all three samples when the voltage deviation is within ±0.2 V from the focal point. Beyond this range, sharpness diminishes more noticeably. Consequently, positions where the normalized sharpness exceeds 0.8 (corresponding to a voltage deviation of ±0.2 V) are defined as "successful."

**Fig. S9 Normalized sharpness curves of the three samples.**

**Table S2 Normalized sharpness values of the three samples near the focal point.**

| **Voltage Error**  **（V）** | **Normalized sharpness value** | | |
| --- | --- | --- | --- |
|  | **Sample 1** | **Sample 2** | **Sample 3** |
| -1.0 | 0.120 | 0.083 | 0.053 |
| -0.9 | 0.147 | 0.099 | 0.075 |
| -0.8 | 0.204 | 0.131 | 0.146 |
| -0.7 | 0.248 | 0.164 | 0.225 |
| -0.6 | 0.324 | 0.218 | 0.390 |
| -0.5 | 0.410 | 0.278 | 0.583 |
| -0.4 | 0.605 | 0.422 | 0.858 |
| -0.3 | 0.723 | 0.526 | 0.920 |
| -0.2 | **0.844** | **0.837** | **0.893** |
| -0.1 | **0.955** | **0.919** | **0.895** |
| 0 | **1.000** | **1.000** | **1.000** |
| 0.1 | **0.953** | **0.985** | **0.943** |
| 0.2 | 0.876 | 0.931 | 0.839 |
| 0.3 | 0.778 | 0.754 | 0.685 |
| 0.4 | 0.585 | 0.625 | 0.476 |
| 0.5 | 0.481 | 0.507 | 0.369 |
| 0.6 | 0.369 | 0.383 | 0.257 |
| 0.7 | 0.295 | 0.303 | 0.191 |
| 0.8 | 0.197 | 0.197 | 0.110 |
| 0.9 | 0.146 | 0.144 | 0.073 |
| 1.0 | 0.120 | 0.083 | 0.053 |

S6: Generalization experiments

To evaluate the performance improvement of the proposed random sampling training method, 1000 autofocus experiments starting from random positions on the same dataset were conducted as shown in Fig. S10. Fig. S10a shows the performance of the DRLAF model trained solely on Sample 1 when applied to Sample 2. In contrast, Fig. S10b illustrates the performance of the model trained on both Sample 1 and Sample 2 using random sampling, also when applied to Sample 2. As illustrated in the figures, the random sampling training method effectively addresses the limitations of deep reinforcement learning models that arise from the homogeneity of the training dataset. By training with a diverse set of sample states, the model acquires a broader range of knowledge, thereby enhancing its autofocus performance across different samples.

**Fig. S10 Generalization improvement of DRLAF by random sampling training. a** Performance of the DRLAF model trained solely on Sample 1 and tested on Sample 2. **b** Performance of the DRLAF trained both Sample 1 and Sample 2 using random sampling and tested on Sample 2. The results demonstrate that random sampling from multiple samples improves the model's autofocus performance on different samples.

Reference

1. Lippmann, G. Relations entre les phénomènes électriques et capillaires. *Ann. Chim. Phys*, 5, 494-549 (1875).
2. Berge, B. Liquid lens technology: principle of electrowetting based lenses and applications to imaging. In *18th IEEE International Conference on Micro Electro Mechanical Systems,* 227-230. IEEE. (Miami Beach, FL, USA, 2005)
3. Brenner J F, Dew B S, Horton J B, et al. An automated microscope for cytologic research apreliminary evaluation|. *Journal of Histochemistry & Cytochemistry Official Journal ofthe Histochemistry Society*, 24, 100-11 (1976).
4. Jarvis R A. Focus optimisation criteria for computer image processing. *Microscope*, 24:163-180 (1976).
5. Maoyon C, Nongliang S & Daoyin Y. Research on sharpness evaluation function of defocusing fuzzy image. *Chinese Journal of Instrument*, z2, 259-260 (2001).
6. Pech-Pacheco, José Luis, et al. Diatom autofocusing in brightfield microscopy: a comparative study. *Proceedings 15th International Conference on Pattern Recognition*, 314-317. IEEE. (Barcelona, Spain, 2000).
